# Chilled Potatoes Decrease Postprandial Glucose, Insulin, and Glucose-dependent Insulinotropic Peptide Compared to Boiled Potatoes in Females with Elevated Fasting Glucose and Insulin

**DOI:** 10.3390/nu11092066

**Published:** 2019-09-03

**Authors:** Mindy A Patterson, Joy Nolte Fong, Madhura Maiya, Stephanie Kung, Araz Sarkissian, Nezar Nashef, Wanyi Wang

**Affiliations:** 1 Department of Nutrition and Food Services, Texas Woman’s University, 6700 Fannin Street, Houston, TX 77030, USA; 2 Office of Research and Sponsored Programs, Texas Woman’s University, 6700 Fannin Street, Houston, TX 77030, USA; 3 Center for Design and Research, Texas Woman’s University, 6700 Fannin Street, Houston, TX 77030, USA

**Keywords:** glucose homeostasis, resistant starch, incretins, subjective satiety, dietary intake

## Abstract

Resistant starch (RS) has been shown to improve postprandial glycemia and insulin sensitivity in adults with metabolic syndrome. RS is found naturally in potatoes, where the amount varies based on cooking method and serving temperature. Thirty females with a mean BMI of 32.8 ± 3.7 kg/m^2^, fasting glucose of 110.5 mg/dL, and insulin of 10.3 µIU/L, completed this randomized, crossover study. A quantity of 250 g of boiled (low RS) and baked then chilled (high RS) russet potatoes were consumed on two separate occasions. Glycemic (glucose and insulin) and incretin response, subjective satiety, and dietary intake were measured. Results showed that the chilled potato elicited significant reductions at 15 and 30 min in glucose (4.8% and 9.2%), insulin (25.8% and 22.6%), and glucose-dependent insulinotropic peptide (GIP) (41.1% and 37.6%), respectively. The area under the curve for insulin and GIP were significantly lower after the chilled potato, but no differences were seen in glucose, glucagon-like peptide-1, and peptide YY, or overall subjective satiety. A higher carbohydrate and glycemic index but lower fat diet was consumed 48-hours following the chilled potato than the boiled potato. This study demonstrates that consuming chilled potatoes higher in RS can positively impact the glycemic response in females with elevated fasting glucose and insulin.

## 1. Introduction

Innovative dietary modifications of commonly consumed foods are needed to combat the rising epidemic of prediabetes. Potatoes are a staple food commodity in many countries and are the third most consumed crop followed by rice and wheat in the United States [1]. Rich in complex carbohydrates, vitamins C and B6, potassium, magnesium, and fiber, while low in fat and sodium, potatoes provide essential nutrients that contribute to the recommended dietary needs of many individuals [2]. 

Starch is the primary carbohydrate found in potatoes and includes both digestible and nondigestible starch, or resistant starch (RS). Resistant starch cannot be broken down by digestive enzymes and enters the large intestine intact. Of the five types of RS, type 2 (RS2) is found in raw and cooked potatoes, while type 3 (RS3) is present in cooked, then chilled potatoes. Environment, natural selection, variety, storage conditions and duration, cooking method, and serving temperature influence the amount of RS in potatoes [3]. Boiled, baked, or microwaved potatoes contain less RS (mean 2.3 g/100 g) than cooked then chilled potatoes (mean 5.6 g/100 g) [4]. Heat and water allow the amylose and amylopectin in the starch granule to disassociate, allowing increased accessibility to digestive enzymes, but chilling reforms the amylose and amylopectin into a packed structure, a process known as retrogradation [5]. 

Resistant starch can influence health-related outcomes through several mechanisms, especially in individuals at risk for developing chronic diseases, including prediabetes. Both RS2 and RS3 can be fermented by gut microbes to produce metabolites such as short-chain fatty acids (SCFA; butyrate, propionate, and acetate), CO_2_, and methane [6]. The metabolites, specifically SCFA, can bind to fatty-acid receptors on the colonocytes to stimulate the release of incretins, such as glucagon-like peptide-1 (GLP-1) [7,8] and glucose-dependent insulinotropic-polypeptide (GIP) [9], as well as peptides known to influence satiety, such as peptide YY (PYY) [7,10]. Attenuated post-prandial glucose [11] and improvements in insulin sensitivity in adults with metabolic syndrome [12,13] have been observed in human trials following RS intake. However, these trials utilized 15–40 g of high-amylose maize RS2 as a functional ingredient or supplement over 4–8 weeks. Different types of RS have demonstrated improvements in satiety and subsequent energy. Improved satiety following RS4 [14], and reduced energy at a subsequent meal after RS2 [15], and over 24-hour following wheat RS [16] intake have been observed. Although these trials did not use RS3 as an intervention, significant reductions in glycemic response have been observed following acute cooked then chilled potato consumption, which would have higher concentrations of RS3—although the amount was not reported—compared to boiled potatoes [17]. Currently, the use of RS3 as a daily intervention of longer duration (≥4 weeks) in clinical trials examining glycemic and satiety outcomes is limited. Randomized-controlled feeding trials of longer duration using RS3 are needed to provide more evidence on the possible glycemic benefits of different types of RS. 

Because potatoes are widely consumed worldwide and are a natural source of RS that can be altered by the cooking method and serving temperature, it is important to identify the potato form that most favorably modulates glycemic response to help combat the progression of chronic disease in overweight and obese adults. Thus, the purpose of this study is to compare glucose, insulin, incretin (GLP-1 and GIP), and a peptide known to influence satiety (PYY) concentrations following the intake of (1) boiled russet potatoes consumed hot and (2) baked then chilled russet potatoes consumed cold, in overweight and obese females with elevated fasting glucose and insulin concentrations. In addition, we examine the relationship among gut-derived peptides, subjective satiety, and 48-hour subsequent energy and nutrient intake following boiled and chilled potato consumption, as well as how body composition may influence glycemic response. We hypothesized that glycemic response would be attenuated following chilled potato intake, which may be augmented by incretin secretion. We also hypothesized that subjective satiety would be higher and 48-hour subsequent energy would be lower following intake of the chilled potato compared to intake of the boiled potato. The rationale behind a reduction in subsequent energy is related to the delayed fermentation of RS which has been shown to occur between 5 to 7 h following intake [18]. The metabolites produced from RS fermentation by the gut microbiota would stimulate the production of incretins and satiety peptides to influence subsequent energy.

## 2. Material and Methods

### 2.1. Study Design

Thirty premenopausal overweight and obese females (BMI ≥ 28–40 kg/m^2^) between 18 and 45 years of age of any ethnicity and race were enrolled in this randomized crossover study. Exclusion criteria included a diagnosis of diabetes or other metabolic disorder, cancer, or cardiovascular disease. Although the inclusion criteria for prediabetic concentrations of fasting glucose and insulin were not a component of the screening procedures, the study results indicated the females had elevated fasting glucose and insulin at baseline. Additional exclusion criteria included nicotine or drug use, females who were pregnant or lactating, and those presenting a significant weight change (≥5% over the prior six months) or following a special diet (e.g., gluten-free, low carbohydrate). Subjects with a sensitivity or aversion to potatoes were excluded. The subjects were recruited from the Texas Medical Center in Houston, Texas, and surrounding areas. 

Subjects were randomized based on a random number generator to receive one of the following on the first visit: 250 g boiled russet potato consumed hot or 250 g baked then chilled russet potato consumed chilled. Body composition (fat and fat-free mass) was assessed using air displacement plethysmography (BOD POD^®^, COSMED USA Inc., Concord, CA, USA) with the subject wearing spandex clothing and a swim cap at the first visit only. Subjects arrived fasted for ≥8 h other than water at the study center on two separate occasions with a minimum of one-week wash-out between visits. Following fasting blood collection, the subjects were given either the boiled or chilled potato based on randomization. A research assistant noted the start and finish time, based on a timer associated with each subject. The subjects consumed their potato within 15 min with 237 mL of water. Additional blood was collected at 15, 30, 60, and 120 min at the completion of potato intake. A 100 mm visual analogue scale (VAS) [19] assessed subjective satiety at 15 and 60 min after the intake of each potato. All timed measurements were logged on the data collection sheet. Food records were completed three days prior to and 48 h following each potato intervention for a total of ten records per subject. The day prior to each intervention, the subject was instructed by a Registered Dietitian Nutritionist to consume a low-fiber (≤10 g) diet. This study was approved by the University Institutional Review Board in Houston, Texas, and all subjects provided informed consent prior to data collection. The trial was registered with the U.S. National Library of Medicine (NCT Clinical trial number NCT0331047).

### 2.2. Potato Preparation

Russet potatoes of similar size were purchased from the same local grocery store prior to each intervention. The boiled potatoes were prepared by washing, peeling the skin, dicing into 2.54 cm cubes, and boiled in water for 15 min until soft. The decision to peel the potato prior to boiling, as well as the duration of boiling time, was to mimic typical preparation methods used when boiling potatoes. The potatoes were drained, measured to the nearest gram, and served to the subject while hot without any additives. The chilled potatoes were washed and wrapped in foil, then baked in a convection oven at 218 °C for 90 min or until soft. The foil was removed, and the potato was placed in a sealed container and stored at 4 °C for five days. The peeling remained on the potato during the baking and chilling process to preserve potato quality, which could have been reduced if the peeling were removed. The potatoes were chilled five days to maximize RS3 formation, which is similar to a protocol used previously [20] where potatoes were chilled for six days. However, due to food safety concerns, the potatoes were chilled five days. Immediately prior to consumption, the skin was removed, the potato was slightly mashed and weighed to the nearest gram, then served to the subject cold without any additives.

### 2.3. Biomarker Analysis

Blood was collected in EDTA (for glucose, insulin, and GIP measurements) and BD P800 (for GLP-1 and PYY measurements) vacutainers, centrifuged at 4000 rpm for 15 min to separate plasma from erythrocytes. Plasma was aliquoted into 1.5 mL cryovials and stored at −80 °C until analyzed by a multi-mode reader (Synergy HI, BioTek^®^ Instruments, Inc., Winooski, Vermont, U.S.A.). Glucose was determined by colorimetric analysis. Insulin, GLP-1(active), GIP (total), and PYY_(1–36 and 3–36)_ were measured using an enzyme-linked immunosorbent assay from commercially prepared kits (Alpco, Salem, New Hampshire, U.S.A.) according to instructions. Calculated intra- and inter-coefficients of variation for all biomarkers were less than 10% and 15%, respectively.

### 2.4. Statistical Analysis

The main outcome was to compare the area under the curve (AUC_(0–120 min)_) glucose between potato interventions (boiled vs. chilled). Based on previous data [10], a large effect size of 0.8 was calculated based on the significant reduction in AUC glucose observed after RS intake. For a more rigorous study design, a more conservative moderate–high effect size of 0.7 was used to calculate the sample size for this study. *A priori* analysis using G × Power 3.1.9 determined a sample size of 29 for a paired-*t* test with a power of 0.95 and alpha of 0.05. The final sample size for this study included 30 subjects. Missing data were treated by pairwise deletion. Descriptive statistics (minimum, maximum, mean, and SD) were calculated for all continuous variables. The AUC_(0–120 min)_ was calculated for each biomarker using the trapezoid formula. Differences between boiled and chilled potatoes at each blood collection time point and the AUC_(0–120 min)_ was determined by a Wilcoxon signed ranks test. For the dietary data, the mean energy and nutrient intake three days prior to each intervention and subsequent 48 h were calculated for each potato intervention. The mean intake between each phase, as well as difference from pre-to-post was compared by Wilcoxon signed ranked test. The relationship among body composition and AUC_(0–120 min)_ biomarkers, subjective satiety within-and-between potato interventions, and subjective satiety with the AUC_(0–120 min)_ for biomarkers known to influence satiety were assessed using Spearman’s rho correlations. SPSS version 25 (Armonk, NY, U.S.A.) was used to perform all analysis. A *p* < 0.05 indicates a significant difference.

## 3. Results

### 3.1. Subjects

**Figure 1:**
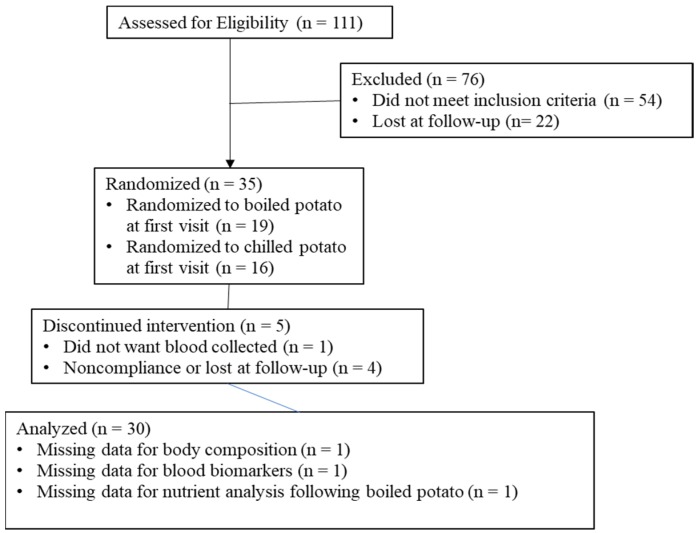
Subject flow through the study protocol.

Data on subject recruitment, enrollment, completion, and analysis can be found in Figure 1. Of the potential subjects expressing interest (*n* = 111), 68.4% completed screening. Thirty-five subjects met inclusion criteria, with 85.7% (*n* = 30) completing the trial. Subject characteristics at baseline can be found in Table 1.

### 3.2. Biomarker Response between Groups

**Figure 2:**
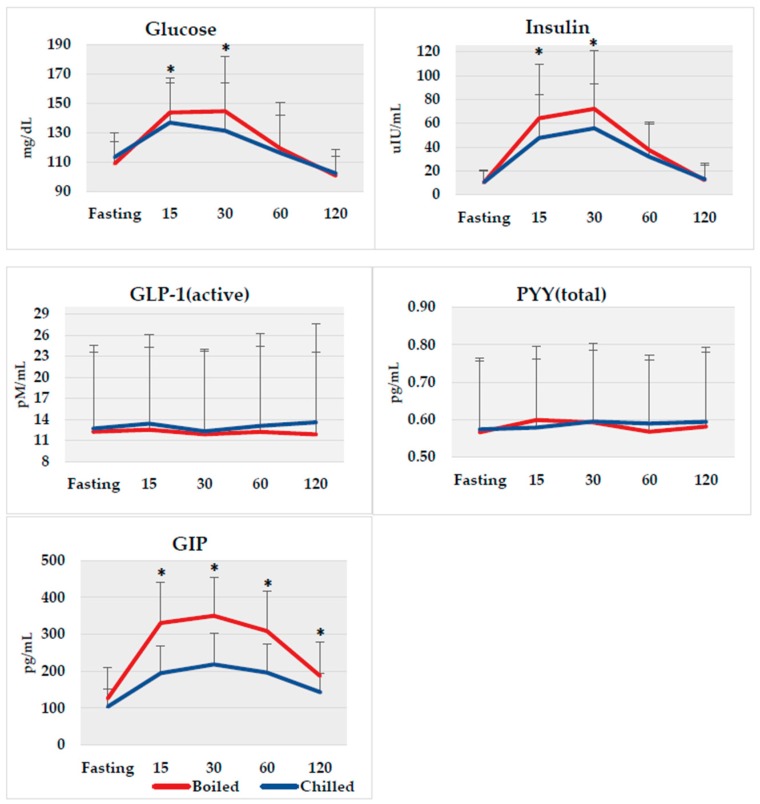
Biomarker responses from fasting to 120 min following boiled (red line) and chilled (blue line) potato intake. * *p* < 0.05 between groups using non-parametric Wilcoxon Signed Ranks Test. GLP-1, glucagon-like peptide-1; PYY, peptide YY; GIP, glucose-dependent insulinotropic-peptide.

On average, our subjects had elevated fasting glucose of 110.5 mg/dL (normal values are <100 mg/dL) and fasting insulin of 10.3 µIU/L (normal values are 3–8 µIU/mL) at baseline. No differences were observed in fasting biomarker concentrations between potato interventions. Glucose, insulin, GLP-1, PYY, and GIP postprandial responses at 15, 30, 60, and 120 min between each potato method are shown in Figure 2. Although the AUC_(0–120 min)_ for glucose did not differ between boiled and chilled potatoes, which was the main outcome, glucose was lower at postprandial times of 15 and 30 min by 4.8% (*p* = 0.03) and 9.2% (*p* = 0.03), respectively, after consuming the chilled potato compared to the boiled potato. Insulin also decreased by 25.8% (*p* = 0.005) at 15 min and 22.6% (*p* = 0.004) at 30 min after intake of the chilled potato compared to the boiled potato. In addition, GIP was significantly lower at 15, 30, 60, and 120 min by 41.1%, 37.6%, 36.4%, and 23.5%, respectively, following chilled potato intake compared to GIP after the boiled potato (all time points *p* < 0.0001). No difference was observed for GLP-1 and PYY at any postprandial time point.

**Figure 3:**
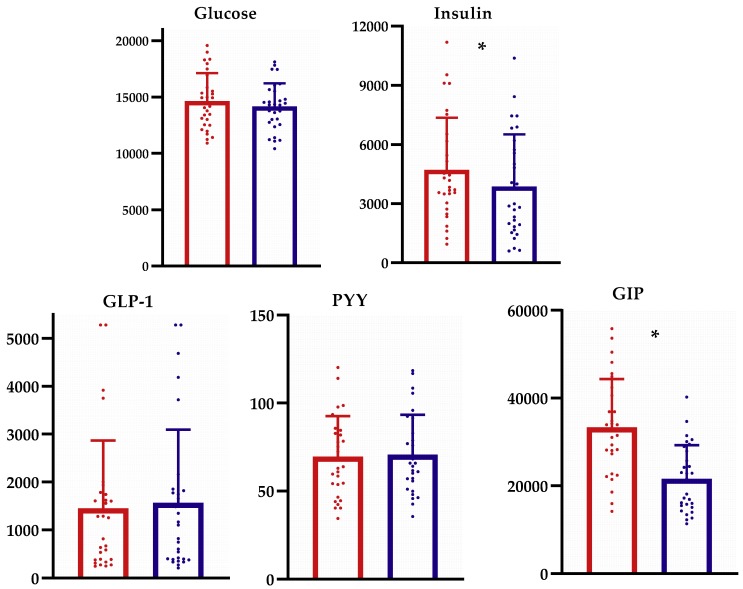
Comparison of the area under the curve (AUC_0–120 min_) biomarker response between boiled and chilled potato intake. * *p* < 0.05 between groups using non-parametric Wilcoxon Signed Ranks Test. Red color refers to the AUC_(0–120 min)_ for the boiled potato; blue color refers to the AUC_(0–120 min)_ for the chilled potato. GLP-1, glucagon-like peptide-1; PYY, peptide YY; GIP, glucose-dependent insulinotropic-peptide.

The AUC_(0–120 min)_ values for each biomarker were compared between potato methods and are displayed in Figure 3. Following chilled potato consumption, the AUC_(0–120 min)_ for insulin and GIP were significantly lower by 17.7% and 35.1%, respectively, compared to those following boiled potato consumption. No difference in the AUC_(0–120 min)_ for glucose, GLP-1, or PYY occurred between groups.

### 3.3. Relationship among Body Composition and Biomarker Response between Interventions

The subjects had a mean BMI of 32.8 ± 3.7 kg/m^2^. Because body composition can influence biomarker response to food, [21] the relationship between body composition and the AUC_(0–120 min)_ for each biomarker was determined (Table S1). Percent fat mass (*r* = 0.425; *p* = 0.024) was weakly positively correlated and fat mass in kg (*r* = 0.387; *p* = 0.042) was moderately positively correlated with insulin AUC_(0–120 min)_ following chilled potato intake. Percent fat-free mass (*r* = −0.425; *p* = 0.024) showed a moderate negative association with insulin AUC_(0–120 min)_ after chilled potato intake. These relationships were not found following boiled potato intake. In addition, fat-free mass in kg (*r* = −0.415; *p* = 0.028) was moderately negatively associated with GIP AUC_(0–120 min)_ following chilled potato intake. However, the correlation coefficient (*r*) between the two groups was not significantly different; all *p* > 0.05.

### 3.4. Dietary Intake Before and after each Intervention

Mean ± standard deviation (SD) in dietary intake was recorded three days prior to and 48-hours following each potato intervention. The comparison of energy and nutrient intake before and after each potato, as well as the difference in change (pre-to-post), can be found in Table 2. No difference in dietary intake was observed before and after boiled potato intake. However, the percentage of kcal from carbohydrates and the intake of foods with a higher glycemic index increased while dietary saturated fat and percentage of kcal from fat decreased following chilled potato intake. When comparing the differences before and after each potato intervention, total, polyunsaturated, and saturated fat intake was less but the percentage of kcal from carbohydrates and foods with a higher glycemic index were increased following chilled potato intake compared to the boiled potato.

### 3.5. Subjective Satiety

Data for subjective satiety, which includes the individual questions and how the scores were interpreted to measure subjective satiety, can be found in Table S2. When comparing each VAS question between time points within each group, the subjects had a 10.9 mm increase in mean score for “How hungry do you feel?” (*p* = 0.026) and 13.6 mm increase in mean score for “How much do you think you can eat?” (*p* = 0.012) from 15 min to 60 min following consumption of the chilled potato. Based on the results of these two questions, the subjects had increased hunger from 15 to 60 min after chilled potato intake. Following intake of the boiled potato, the subjects scored 9 mm (*p* = 0.028) higher for “How pleasant would you find eating another mouthful of this food?” at 60 min compared to 15 min, suggesting they would find eating another bite pleasant.

When comparing VAS questions between potato interventions, the question “How pleasant would you find eating another mouthful of this food?” scored 10.7 mm higher (*p* = 0.028) after the boiled potato at 60 min postprandial than the chilled potato. These results indicate the chilled potato was less palatable than the boiled potato 60 min after consumption.

When comparing the gut-derived biomarkers to subjective satiety (Table S3), the AUC_(0–120 min)_ GLP-1 was positively related (*r* = 0.369, *p* = 0.049) to the mean score for “Would you like to eat something savory?” at 60 min following consumption of the boiled potato. No other gut-derived biomarkers were related to subjective satiety scores, which is an expected finding because the fermentation of RS is not likely to occur during this time frame.

## 4. Discussion

This study examined the impact of RS from russet potatoes on glucose metabolism, incretin secretion, and dietary intake in females with elevated fasting glucose and insulin. This study is novel in that it compared how cooking and serving methods modified RS amount from a natural, commonly consumed food on glycemic response. Although the AUC_(0–120 min)_ glucose did not differ between groups, which was the primary outcome, the chilled potato reduced mean glucose and insulin in the immediate (15 and 30 min) postprandial period, as well as the AUC_(0–120 min)_ for insulin.

Similar to our findings, Ma et al. [22] found, in obese adults, a significant reduction in serum glucose 30 min following the consumption of 30 g RS from a muffin when compared to a glucose solution with equivalent digestible carbohydrate. Another study also examined glycemic and insulinemic responses in healthy adults following 40 g native banana starch beverage containing 28.2 g RS2 versus a 40 g digestible corn starch beverage, noting a significant reduction in glucose between 30 and 120 min and insulin between 60 and 90 min following the banana starch [23]. This study matched the total amount of beverage administered, not digestible carbohydrate, which is similar to the present study where the amount of potato (250 g) consumed was matched. Another study found a reduction in glucose at 15, 45, and 60 min following the intake of cooked rice that was cooled for 10 h compared to cooked rice in healthy adults [24]. The rice would have contained RS3 due to retrogradation of the starch granule from the cooling process, which is the type of RS found in the chilled potatoes in our study. Another study examining the glycemic index among potatoes matched for available carbohydrate found a reduction in the AUC glucose, which was not observed in the present study. Fernandes et al. [25] found that boiled red potatoes chilled between 12 and 24 h produced a larger reduction in the AUC glucose (135 mmol x min/L) than boiled red potatoes consumed hot (208 mmol x min/L) [25]. However, a different type of potato was used, both males and females were included, the subjects had a lower mean BMI (22.3 kg/m^2^), and the duration of the potato chilling process differed from the present study. These differences in study design may partially explain why a reduction in the AUC_(0–120 min)_ following chilled potato intake did not differ from the values associated with boiled potato intake in the present study.

In the present study, the reduction in postprandial glucose at 15 and 30 min and the AUC_(0–120 min)_ insulin following chilled potato intake may be partially explained by GIP secretion, which was also lower at all postprandial time points, and the AUC_(0–120 min)_ following chilled potato intake. Glucose stimulates GIP release, which peaks within 5 min of glucose intake compared to GLP-1 release that peaks at 30 min [26]. Both GIP and GLP-1 have insulinotropic effects on pancreatic β-cells but are secreted in different sections in the gastrointestinal (GI) tract: GIP from the duodenum and GLP-1 from the distal ileum and colon [27,28]. The chilled potato contains more RS, which displaces the amount of available carbohydrate when compared to the boiled potato. Thus, less glucose is available, which was observed after the chilled potato at 15 and 30 min postprandially, to stimulate GIP in early digestion. The differences in the available carbohydrate between potatoes, not the production of SCFA from the fermentation of RS in the distal GI, is likely attributed to these results —especially, since GLP-1 concentrations were not different between potatoes. However, in mice, RS reduced GIP mRNA in the jejunum in a dose-dependent manner [28]. Other studies have found similar results to the present study regarding incretins, peptides that induce satiety, and VAS-measured subjective satiety [19]; but some studies have reported improvements in GLP-1 following RS intake [29]. Similar to GLP-1, no difference in PYY concentrations occurred between groups.

Interestingly, a higher insulin response was associated with the subjects who had a higher fat mass but lower fat-free mass following intake of the chilled potato but not the boiled potato (Table S1). This phenomenon may be due to metabolic inflexibility, where the subjects with a less favorable body composition were not able to physiologically adapt following the intake of the potato with less available carbohydrate [30]. Going from a fasted to fed state, or insulin-stimulating state, in a metabolic inflexibly environment contributes to insulin resistance by reducing glucose oxidation [31]. Skeletal muscle, which constitutes the majority of fat-free mass, plays a role in metabolic flexibility in that it accounts for 60–80% of glucose uptake as a result of insulin stimulation [32]. In our subjects, those with less fat-free mass demonstrated higher insulin concentrations when less carbohydrate was available, signifying a reduction in glucose oxidation.

Despite differences in RS between the boiled and chilled potatoes, an increase in subjective hunger was reported from 15 to 60 min following the chilled potato only. This result would not be related to the fermentation of RS to produce SCFA to stimulate the release of gut peptides GLP-1 and PYY that could induce immediate postprandial satiety. The fermentation of RS depends on gut transit time [33], which typically occurs between 5 and 7 h following RS intake [18], and would, therefore, not be captured within the 2 h window of subjective satiety measurement. Instead, the increased subjective hunger may be related to palatability. The subjects reported the chilled potato to be less pleasing or palatable from 15 to 60 min following consumption, which remained lower than the boiled potato at 60 min. Obese individuals have shown higher hedonic hunger scores compared to non-obese individuals, which are inversely associated with glycemic control [34]. Owing to a combination of the reduction in palatability and lower insulin levels, the chilled potato may have caused an increased perception of hunger with a decreased stimulus from insulin in the reward center of the brain [35]. Additionally, a higher dose of RS may be needed to see a significant change in subjective satiety scores [29]. The type of RS may also impact satiety, where one study found RS3 supplement had lower satiety scores than an RS2 supplement [36].

Following the chilled potato intervention, an increased percentage of energy from carbohydrates and higher glycemic foods were consumed when compared to intake prior to the intervention, as well as the change from pre-to-post following the boiled potato. The carbohydrate foods displaced dietary fat while energy stayed the same. The lower amount of available carbohydrate consumed with the chilled potato could have promoted an increase in carbohydrate-rich foods. According to a review of RS on energy intake and subsequent, subjective satiety in healthy subjects [37], the majority of studies found no differences in VAS scores and mixed results for total energy intake following RS consumption [35,36,37,38,39,40].

The present study has several strengths. A commonly consumed, natural food product was used for the intervention. The amount of potato administered was approximately 8 ounces, or 1 cup, which is an amount that could be consumed in one meal. In addition, a realistic dose of RS found naturally in potatoes was used in the intervention, whereas most clinical trials use much higher doses of RS (~15–40 g/day) as a supplement in the intervention. The randomized, crossover study design allowed for a sufficient sample (*n* = 30), while minimizing the confounding variables. The present study also analyzed dietary intake over a 10-day period (3 days prior to and 48 h following each intervention) to include weekdays and weekends that most likely represented habitual eating patterns.

Several limitations should also be noted. The study was not blinded due to the differences in potato intervention (chilled vs. hot). Only females were included, so the findings cannot be extrapolated to other populations and disease states. Also, although the potatoes were purchased from the same local grocery store, growing and storage conditions were not controlled which could impact RS concentration [3].

## 5. Conclusions

Chilled potatoes with a higher concentration of RS, thus less available carbohydrate, are effective in reducing postprandial glucose, insulin, and GIP, when compared to boiled potatoes consumed hot. A less favorable body composition resulted in a less favorable insulinemic response after chilled potato intake. The chilled potato did not induce satiety, it was less palatable, and it did not alter subsequent energy intake when compared to the boiled potato. Altering the cooking method and serving temperature of this staple food has the potential to improve glycemic indices in females with elevated fasting glucose and insulin.

## Figures and Tables

**Table 1:** Subject characteristics (*n* = 30).

Variables	Mean ± Standard Deviation (SD)
Age (years)	29.6 ± 6.0
Body weight (kg)	85.6 ± 11.8
BMI (kg/m^2^)	32.8 ± 3.7
Percent fat mass	43.6 ± 4.9
Percent fat-free mass	56.4 ± 4.9
Fat mass (kg)	37.9 ± 8.0
Lean mass (kg)	48.5 ± 5.1

**Table 2:** Comparison of mean nutrient intake before and after each potato intervention.

Variable	Mean ± SD Boiled	*p*-Value Boiled	Mean ± SD Chilled	*p*-Value Chilled	*p*-Value Difference in Change between Groups
**Energy (kcal)**					
Pre	1828 ± 842	0.329	1933 ± 891	0.528	
Post	1987 ± 1223		1843 ± 1220		
Difference	160 ± 865		−67 ± 774		0.185
**CHO (g)**					
Pre	211.2 ± 95.9	0.55	206.0 ± 83.8	0.457	
Post	223.9 ± 140.6		218.6 ± 130.3		
Difference	12.7 ± 112.7		16.1 ± 90.8		0.876
**Protein (g)**					
Pre	76.6 ± 45.2	0.753	77.7 ± 34.7	0.895	
Post	78.5 ± 49.5		76.8 ± 52.8		
Difference	1.9 ± 32.0		−1.2 ± 37.2		0.72
**Fat (g)**					
Pre	76.6 ± 40.5	0.328	89.5 ± 55.6	0.065	
Post	84.5 ± 60.4		75.1 ± 62.2		
Difference	7.9 ± 42.8		−13.2 ± 41.4		**0.026**
**Monounsaturated fat (g)**					
Pre	27.7 ± 15.7	0.608	31.9 ± 18.6	0.095	
Post	28.9 ± 19.0		26.9 ± 22.7		
Difference	1.2 ± 12.9		−4.5 ± 16.0		0.09
**Polyunsaturated fat (g)**					
Pre	17.6 ± 9.7	0.294	22.0 ± 15.0	0.11	
Post	20.2 ± 16.7		18.1 ± 14.2		
Difference	2.6 ± 13.3		−3.9 ± 13.4		**0.013**
**Saturated fat (g)**					
Pre	24.8 ± 13.6	0.303	28.5 ± 20.6	**0.047**	
Post	28.2 ± 21.2		23.7 ± 21.6		
Difference	3.4 ± 17.6		−4.2 ± 12.5		**0.045**
**Trans fatty acids (g)**					
Pre	2.3 ± 1.8	0.679	2.4 ± 1.6	0.608	
Post	2.2 ± 1.3		2.6 ± 2.9		
Difference	−0.1 ± 1.6		0.3 ± 2.7		0.345
**% kcal from CHO**					
Pre	46.4 ± 9.3	0.488	44.0 ± 8.7	**0.033**	
Post	45.3 ± 10.7		49.3 ± 11.7		
Difference	−1.1 ± 8.7		5.4 ± 13.0		**0.032**
**% kcal from protein**					
Pre	16.6 ± 5.1	0.875	16.8 ± 4.6	0.688	
Post	16.4 ± 4.6		16.4 ± 5.3		
Difference	−0.2 ± 5.6		−0.7 ± 5.4		0.692
**% kcal from fat**					
Pre	36.5 ± 6.6	0.798	38.3 ± 6.8	**0.032**	
Post	36.2 ± 8.7		33.9 ± 9.1		
Difference	−0.4 ± 7.6		−4.3 ± 11.0		0.088
**Fiber (g)**					
Pre	14.8 ± 7.0	0.502	15.6 ± 6.2	0.418	
Pro	15.8 ± 10.2		16.7 ± 8.9		
Difference	1.1 ± 8.3		1.5 ± 7.0		0.813
Glycemic index *					
Pre	59.8 ± 4.5	0.833	60.2 ± 4.0	**0.001**	
Post	60.0 ± 3.7		64.3 ± 7.3		
Difference	0.2 ± 5.9		4.1 ± 6.5		**0.049**
**Glycemic load**					
Pre	118.2 ± 52.8	0.539	115.5 ± 49.0	0.171	
Post	125.1 ± 78.3		130.0 ± 76.9		
Difference	6.9 ± 59.8		15.3 ± 52.6		0.486

* Glycemic index was calculated using glucose as the reference. The bold shows the significant differences.

## Supplementary Materials

The following are available online at https://www.mdpi.com/2072-6643/11/9/2066/s1, Table S1: Correlations among body composition and the area under the curve (AUC) for biomarker responses following boiled and chilled potato intake ^1,2^, Table S2: Comparison of within-and-between group mean subjective satiety scores measured at 15 and 60 min following boiled and chilled potato intake, Table S3: Correlations between the area under the curve for biomarkers known to influence satiety and mean subjective satiety scores between potato interventions ^1,2^.
