# Supplementary material for: Chilled Potatoes Decrease Postprandial Glucose, Insulin, and Glucose-dependent Insulinotropic Peptide Compared to Boiled Potatoes in Females with Elevated Fasting Glucose and Insulin
Source: Nutrients. 2019 Sep 3;11(9):2066. doi: 10.3390/nu11092066 (PMC6769955; doi:10.3390/nu11092066)
Supplement: Supplementary file 1 [file nutrients-11-02066-s001.pdf]

Table 1. Correlations among body composition and the area under the curve (AUC) for biomarker responses following boiled and chilled potato intake[1,2].

Variable	$AUC_{(0-120\ min)}$ Glucose[1]	$AUC_{(0-120\ min)}$ Insulin[1]	$AUC_{(0-120\ min)}$ GLP-1[1]	$AUC_{(0-120\ min)}$ PYY[1]	$AUC_{(0-120\ min)}$ GIP[1]
**Boiled Potato**					
BMI (kg/m^2)	0.121	0.224	-0.242	-0.194	-0.217
% FM	0.107	0.343	-0.083	0.048	-0.075
% FFM	-0.107	-0.343	0.083	-0.048	0.075
FM (kg)	0.074	0.343	-0.242	-0.068	-0.181
FFM (kg)	-0.142	0.054	-0.306	-0.204	-0.360
**Chilled Potato**					
BMI (kg/m^2)	0.121	0.290	-0.239	-0.200	-0.322
% FM	0.135	**0.425***	-0.124	0.085	0.069
% FFM	-0.135	**-0.425***	0.124	-0.085	-0.069
FM (kg)	0.041	**0.387***	-0.297	-0.007	-0.123
FFM (kg)	-0.117	0.163	-0.235	-0.260	**-0.415***

[1] Correlations determined by nonparametric Spearman's rho. [2]No significant differences were observed using paired t-tests between groups after transforming *r* to *z*; $p > .05$. *$p < 0.05$. AUC = area under the curve; BMI = body mass index; FM = fat mass; FFM = fat-free mass; GIP = glucose-dependent insulinotropic peptide; GLP-1 = glucagon-like peptide-1; PYY = peptide YY

Table 2. Comparison of within-and-between group mean subjective satiety scores measured at 15 and 60 minutes following boiled and chilled potato intake.

Question[1]	Boiled Potato	Chilled Potato	*p* value between group at either 15 or 60 min**
1. How hungry do you feel?[a]			
15 minutes	24.4 ± 29.1	**24.1 ± 27.1**	0.960
60 minutes	27.6 ± 29.4	**34.6 ± 32.0***	0.278
2. How satisfied do you feel?[b]			
15 minutes	67.8 ± 27.8	61.0 ± 31.2	0.234
60 minutes	57.7 ± 31.6	50.4 ± 33.4	0.234
3. How full do you feel?[c]			
15 minutes	64.9 ± 30.0	59.2 ± 32.7	0.227
60 minutes	58.8 ± 30.5	50.5 ± 35.4	0.209
4. How much do you think you can eat?[d]	37.4 ± 30.0	**31.2 ± 29.8**	0.242
15 minutes	42.6 ± 31.9	**44.8 ± 31.7***	0.661
60 minutes			
5. How pleasant would you find eating another mouthful of this food?[e]			
15 minutes	**27.4 ± 26.9**	27.1 ± 34.9	0.976
60 minutes	**36.4 ± 29.8***	25.7 ± 32.6	**0.028**
6. Would you like something sweet?[f]			

15 minutes	38.1 ± 35.4	46.1 ± 37.9	0.194
60 minutes	46.5 ± 34.8	37.8 ± 33.7	0.224
7. Would you like to eat something			
fatty?[g]	31.8 ± 35.7	31.5 ± 31.9	0.954
15 minutes	39.6 ± 36.9	33.1 ± 31.0	0.329
60 minutes			
8. Would you like to eat something savory?[h]			
15 minutes	46.7 ± 38.5	43.7 ± 36.0	0.728
60 minutes	51.0 ± 38.3	46.9 ± 35.9	0.595

[1] Subjective satiety was measured by drawing a line from 0mm to 100mm. The lines were measured by the investigator in mm by a ruler to obtain the score. The overall mean score was calculated by taking the mean of the 8 questions. [a–i] A higher score indicates the following: [a] = never been more hungry; [b] = cannot eat another bite; [c] = totally full; [d] = I can eat a lot; [e] = very pleasant; [f] = yes very much; [g] = yes very much; [h] = yes very much. *Indicates $p < 0.05$ within groups from 15 to 60 minutes. **Indicates $p < 0.05$ between groups at either 15 minutes or 60 minutes.

Table 3. Correlations between the area under the curve for gut-derived biomarkers and mean subjective satiety scores between potato interventions.[1,2].

Variable	$AUC_{(0-120\ min)}$ GLP-1	$AUC_{(0-120\ min)}$ PYY	$AUC_{(0-120\ min)}$ GIP
**Boiled Potato**			
1. How hungry do you feel?[a]			
15 min	-0.046	0.123	0.119
60 min	0.085	0.029	-0.095
2. How satisfied do you feel?[b]			
15 min	0.012	-0.153	-0.267
60 min	-0.214	-0.092	-0.035
3. How full do you feel?[c]			
15 min	-0.012	-0.146	-0.339
60 min	-0.108	-0.086	0.012
4. How much do you think you can eat?[d]			
15 min	-0.059	0.076	0.171
60 min	0.208	0.055	-0.034
5. How pleasant would you find eating another mouthful of this food?[e]			
15 min	-0.137	-0.289	-0.037
60 min	0.206	-0.197	0.020
6. Would you like to eat something sweet?[f]			
15 min	-0.147	0.013	-0.143
60 min	0.103	0.006	-0.053
7. Would you like to eat something fatty?[g]			
15 min	-0.132	0.030	-0.054
60 min	0.105	0.122	0.144
8. Would you like to eat something savory?[h]			
15 min	-0.069	-0.164	0.077
60 min	**0.369***	0.035	0.138
**Chilled Potato**			

1. How hungry do you feel?[a]			
15 min	-0.048	-0.037	0.070
60 min	-0.052	-0.068	0.158
2. How satisfied do you feel?[b]			
15 min	-0.247	-0.350	-0.361
60 min	0.125	0.051	0.005
3. How full do you feel?[c]			
15 min	-0.058	-0.073	-0.144
60 min	0.002	0.074	-0.145
4. How much do you think you can eat?[d]			
15 min	0.134	0.196	-0.038
60 min	-0.030	-0.001	0.150
5. How pleasant would you find eating another mouthful of this food?[e]			
15 min	0.142	0.053	-0.220
60 min	0.213	0.095	-0.056
6. Would you like to eat something sweet?[f]			
15 min	-0.011	0.260	0.193
60 min	0.093	-0.095	0.050
7. Would you like to eat something fatty?[g]			
15 min	-0.146	0.139	-0.014
60 min	-0.294	0.043	-0.075
8. Would you like to eat something savory?[h]			
15 min	-0.001	0.272	0.169
60 min	-0.151	0.020	0.000

[1] Correlations determined by nonparametric Spearman's rho. [2] Subjective satiety was measured by drawing a line from 0mm to 100mm. The lines were measured by the investigator in mm by a ruler to obtain the score. [a–i] A higher score indicates the following: [a] = never been more hungry; [b] = cannot eat another bite; [c] = totally full; [d] = I can eat a lot; [e] = very pleasant; [f] = yes very much; [g] = yes very much; [h] = yes very much
$^{*}p < 0.05$. AUC = area under the curve; GIP = glucose-dependent insulinotropic peptide; GLP-1 = glucagon-like peptide-1; PYY = peptide YY
